# Prevalence of nonalcoholic fatty liver disease and the related risk factors among healthy adults: A cross-sectional study in Chongqing, China

**DOI:** 10.3389/fpubh.2023.1127489

**Published:** 2023-03-30

**Authors:** Lingxi Kong, Yang Yang, Haidong Li, Youlan Shan, Xin Wang, Xuefeng Shan

**Affiliations:** ^1^Department of Pharmacy, The First Affiliated Hospital of Chongqing Medical University, Chongqing, China; ^2^Department of Anesthesiology, The First Affiliated Hospital of Chongqing Medical University, Chongqing, China; ^3^Foreign Affairs Department of Scientific Research, Stomatological Hospital of Chongqing Medical University, Chongqing, China; ^4^Department of Infectious Disease, The Second Affiliated Hospital of Chongqing Medical University, Chongqing, China; ^5^West China School of Public Health, Sichuan University, Chengdu, China

**Keywords:** nonalcoholic fatty liver disease (NAFLD), prevalence, risk factors, obesity, central obesity

## Abstract

**Background:**

Epidemiological characteristics of nonalcoholic fatty liver disease (NAFLD) in Chongqing, a west-central city of China, remain unclear. The objective of this study was to investigate the prevalence of NAFLD and the related risk factors among healthy adults for physical examination in Chongqing.

**Methods:**

A total of 110,626 subjects were enrolled in the present study. Each of the participants underwent physical examination, laboratory measurements, and abdominal ultrasonography. The chi-square test was employed to compare differences in the NAFLD prevalence, and logistic regression analysis was used to estimate the odds ratio for risk factors of NAFLD.

**Results:**

The prevalence of NAFLD in individuals in the population of Chongqing was 28.5%, and the prevalence in men (38.1%) was significantly higher than that in women (13.6%) (OR = 2.44; 95% CI: 2.31–2.58). NAFLD was more common in men aged 51–60 years and women over 60 years. Approximately 79.1% of the people with obesity and 52.1% of the people with central obesity had NAFLD. The prevalence of NAFLD in people with hypertension and cholelithiasis was 48.9 and 38.4%, respectively. Logistic regression showed that gender, age, body max index (BMI), central obesity, hypertension, impaired fasting glucose/diabetes mellitus (DM), triglyceride (TG), low-density lipoprotein cholesterol (LDL-C), high-density lipoprotein cholesterol (HDL-C), hyperuricemia (HUA), alanine transaminase (ALT), and cholelithiasis were independently associated with the presence of NAFLD.

**Conclusion:**

The prevalence of NAFLD among healthy adults in Chongqing was high. To improve the prevention and management of NAFLD, special attention should be paid to the factors associated with the presence of NAFLD, including higher BMI, higher waist circumference, higher blood glucose, hypertension, hypertriglyceridemia, hyperuricemia, cholelithiasis, and elevated ALT.

## Background

Nonalcoholic fatty liver disease (NAFLD), defined as a chronic disorder of the liver with the presence of lipid accumulation in more than 5% of hepatocytes of nonalcohol users, has developed into the most common cause of liver disease (1). Along with the rising prevalence of obesity, particularly in developed countries, NAFLD is becoming an increasing concern and a huge burden of disease for many countries (2).

Thus far, the exact pathogenic mechanism of NAFLD has not been fully explained. However, as has been stressed, inflammation, metabolic stress, and fibrosis probably might take part in the key processes (3). Approximately 10–20% of patients with NAFLD can progress to nonalcoholic steatohepatitis (NASH), and in the end, 3–5% can progress to cirrhosis (4). It has been reported that 2–3% of NASH and 3.78% of cirrhosis developed into hepatocellular carcinoma (HCC) (5, 6).

Evidence has found that NAFLD is strongly associated with overweight or obesity, and it is also correlated with a higher risk of serious extrahepatic diseases, such as cardiovascular disease (CVD), diabetes, and some other metabolic diseases (7).

The prevalence of NAFLD has been found to be around 25.2% globally, and it is also noted that the Middle East and South America have the highest prevalence of 32 and 30.5%, respectively, while Africa has the lowest prevalence of 13.5% (8, 9). In line with the economic spike and change in lifestyles, within a decade in China, the population affected by NAFLD has soared from 18 to 29% (10). Moreover, with the youngest median age of NAFLD worldwide, China will have to face the impact of advanced complications of the disease in the later decades (11).

Obtaining the epidemiological features of NAFLD in China in order to adopt comprehensive treatment measures is around the corner. Although some epidemiological research concerning NAFLD has been reported in China, so far, no data have been reported about the prevalence of NAFLD in regions in the west-central part of China, which may be different from other areas due to the different economic status and living habits. In the present research, we conducted a cross-sectional study with more than 110 thousand subjects to investigate the prevalence and the major risk factors of NAFLD in the population of Chongqing, a west-central city of China.

## Methods

### Study population

Participants in the present study were selected from healthy adults who took the physical examination in the Health Management Center of a Grade 3 Hospital in Chongqing, west-central of China, from January 1, 2016 to December 31, 2020. Those who participated in the study were individuals who volunteered to do the check-ups or employees who were required to undergo a routine physical examination by their employers. People were excluded for the following reasons: (1) failed to perform abdominal ultrasound (US) examination; (2) had excess alcohol consumption (140 g/week for men and 70 g/week for women); (3) had a history of viral hepatitis, autoimmune hepatitis, malignant liver cancer, or other forms of serious chronic liver disease; (4) did not have complete information. A total of 110,626 subjects were enrolled in the final analysis (information loss of the subjects was 8.97% for weight and height, 9.05% for waist circumference, 8.65% for blood pressure, 1.56% for glucose level, and 1.16% for lipid level). The study protocol was approved by Hospital Ethics Committee of the First Affiliated Hospital of Chongqing Medical University. Written informed consent for participation was not required for this study in accordance with the national legislation and the institutional requirements.

### Anthropometric measurements

Information on medical histories was collected, and anthropometric measurements were done by trained doctors and nurses. The weight and height of all examinees were measured using an electric health analyzer (SK-X80, Shuangjia, China), with the patients lightly clothed and wearing no socks. Body mass index (BMI) was calculated as weight (kg) divided by height (m) squared. Systolic blood pressure (SBP) and diastolic blood pressure (DBP) were obtained in the left arm of patients with a wrist sphygmomanometer (HBP-9021, Omron Healthcare, Japan) in the sitting position. The waist circumference (WC) was measured midway between the lowest ribs and the iliac crest with an unstretchable tape measure.

### Biochemical measurements

All blood samples were drawn in the morning after an at least 8-h overnight fast. Fasting blood glucose (FBG), total serum cholesterol (TC), triglyceride (TG), low-density lipoprotein cholesterol (LDL-C), high-density lipoprotein cholesterol (HDL-C), blood uric acid (UA), alanine transaminase (ALT), aspartate aminotransferase (AST), serum total bilirubin (TBIL), and serum direct bilirubin (DBIL) were detected using a Hitachi 7600 Automatic Biochemical Analyzer (Hitachi, Japan).

### Ultrasound examination

Ultrasound examination was carried out by a trained ultrasonographist who was unaware of the study design using a Diagnostic Ultrasound System and Transducers (EPIQ 7, Philips, USA) with a 3.5-MHz probe.

Fatty liver was diagnosed according to the ultrasound criteria suggested by the Chinese Liver Disease Association (12), which was defined with the following ultrasound features: (1) diffuse enhancement of near-field echo in the hepatic region and gradual attenuation of the far-field echo; (2) unclear display of intrahepatic lacuna structure; (3) mild-to-moderate hepatomegaly with a round and blunt border; (4) color Doppler ultrasonography revealing a reduction in hepatic blood flow signal or is difficult to display, while intrahepatic vessels are not unusual.

Cholelithiasis was diagnosed according to the ultrasound criteria as follows (13): (1) The presence of hyperechoic area in the gallbladder cavity accompanied by an acoustic shadow, which moves along the direction of gravity when changing body position; (2) strong light mass in the common bile duct accompanied by an acoustic shadow and bile duct dilation of the proximal liver.

### Definition of variables

**BMI**: Body mass index of <18.5 kg/m^2^ is defined as underweight, BMI of 18.5–23.9 kg/m^2^ as normal weight, BMI of 24–27.9 kg/m^2^ as overweight, and BMI of ≥28 kg/m^2^ as obesity according to the guidelines for prevention and control of overweight and obesity in Chinese adults (14).

**Central obesity**: Central obesity was defined as a waist circumference (WC) ≥ 85 cm for men and ≥80 cm for women (15).

**Hypertension:** Hypertension is defined as systolic blood pressure (SBP) ≥ 140 mmHg and/or diastolic pressure (DBP) ≥ 90 mmHg (1 mm Hg = 0.133 kPa) or patients having been diagnosed with hypertension and receiving treatment, according to Chinese Guidelines for the Management of Hypertension (2010) (16).

**Glucose status**: Normal blood glucose and impaired fasting glucose (IFG) were defined as FBG < 6.1 mmoL and 6.1 mmol/L ≤ FBG < 7.0 mmol/L, respectively. Diabetes mellitus (DM) was defined as FBG ≥ 7.0 mmol/L or under treatment (17).

**Dyslipidemia:** The diagnosis of dyslipidemia was based on the definition recommended by Chinese guidelines for the management of dyslipidemia in adults (18). Hypertriglyceridemia: triglycerides (TC) ≥ 1.7 mmol/L; hypercholesterolemia (TC): cholesterol ≥ 5.2 mmol/L; low HDL-C: HDL-C < 0.9 mmol/L; high LDL-C: LDL-C ≥ 3.1 mmol/L.

**Hyperuricemia:** Hyperuricemia (HUA) was defined as serum uric acid ≥ 416 μmol/L in men and ≥357 μmol/L in women.

**Liver function:** Elevated ALT and Elevated AST were defined as >40 IU/L; elevated TBIL was defined as >17.1 μmol/L; elevated DBIL was defined as >6.8 μmol/L.

### Statistical Analysis

Statistical analysis was conducted using the SPSS software 22.0 (IBM, SPSS Inc., USA). Continuous variables were expressed as mean and standard deviation (SD) and compared with Student's *t*-test or the Mann–Whitney *U*-test. Categorical variables were expressed as percentages or counts and compared with the chi-squared (χ^2^) test. Univariate binary logistic regression analysis was applied to evaluate the factors associated with the presence of NAFLD. The variables with a *P*-value of <0.05 were then included in multivariable binary logistic regression analysis to assess the independent risk factors of NAFLD. All tests were two-tailed, and a *P-*value of <0.05 was considered to be statistically significant.

## Results

### Baseline features of general population

Overall, 110,626 subjects were enrolled in the study, of which 67,256 were men and 43,370 were women. As we can draw from Table 1, 31,535 of the entire people were diagnosed with NAFLD, with a prevalence of 28.5%. The prevalence rate of NAFLD in the male population (38.1%, *N* = 25,624) was significantly higher than that of the female population (13.6%, *N* = 5,911) (χ^2^ = 7,746.839, *P* < 0.001), indicating that NAFLD was 2.8 times more common in men than women. The average age in the NAFLD group (45.06 ± 12.54) was significantly higher than that of the non-NAFLD group (41.48 ± 13.42) (*P* < 0.001) (Table 1). When compared to the non-NAFLD group, subjects in the NAFLD group had a significantly higher level of BMI, WC, SBP, DBP, FBG, TC, TG, LDL-C, UA, ALT, AST, and DBIL and lower levels of HDL-C (*P* < 0.001) (Table 1). When divided by gender, both in the male and female groups, age, BMI, WC, SBP, DBP, FBG, TC, TG, LDL-C, HDL-C, UA, ALT, AST, DBIL, and TBIL for the NAFLD population were significantly different from that of the non-NAFLDs (*P* < 0.001).

**Table 1:** Baseline characteristics of the subjects (*N* = 110,626).

	**Total**				**Male**				**Female**			
	**Non-NAFLD**	**NAFLD**	χ^2^**/t**	***P***	**Non-NAFLD**	**NAFLD**	χ^2^**/t**	***P***	**Non-NAFLD**	**NAFLD**	χ^2^**/t**	***P***
Total	79,091 (71.5)	31,535 (28.5)			41,632 (61.9)	25,624 (38.1)			37,459 (86.4)	5,911 (13.6)		
Age (years)	41.48 ± 13.42	45.06 ± 12.54	−40.747	< 0.001	42.14 ± 14.08	43.49 ± 11.96	−12.768	<0.001	40.74 ± 12.61	51.83 ± 12.75	−62.729	<0.001
Age (years)			2,475.505	<0.001			1,400.05	<0.001			3,771.153	<0.001
≤ 30	19,001 (83.0)	3,902 (17.0)			9,828 (73.5)	3,545 (26.5)			9,173 (96.3)	357 (3.7)		
31–40	23,750 (72.8)	8,873 (27.2)			12,126 (60.3)	7,996 (39.7)			11,624 (93.0)	877 (7.0)		
41–50	16,460 (67.4)	7,961 (32.6)			8,428 (55.4)	6,785 (44.6)			8,032 (87.2)	1,176 (12.8)		
51–60	11,479 (62.7)	6,818 (37.3)			6,049 (55.3)	4,899 (44.7)			5,430 (73.9)	1,919 (26.1)		
>60	8,401 (67.8)	3,981 (32.2)			5,201 (68.4)	2,399 (31.6)			3,200 (66.9)	1,582 (33.1)		
BMI (Kg/m^2^)	22.41 ± 3.21	26.26 ± 4.82	−125.496	<0.001	23.16 ± 2.97	26.39 ± 4.81	−104.086	<0.001	21.52 ± 3.25	25.65 ± 4.83	−78.623	<0.001
WC (cm)	78.11 ± 8.78	89.76 ± 7.51	−198.796	<0.001	82.49 ± 7.32	90.97 ± 6.90	−144.194	<0.001	72.91 ± 7.42	84.13 ± 7.70	−100.167	<0.001
SBP (mmHg)	120.47 ± 16.44	131.30 ± 17.18	−93.7	<0.001	124.02 ± 15.49	131.00 ± 16.33	−53.78	<0.001	116.26 ± 16.53	132.69 ± 20.62	−64.069	<0.001
DBP (mmHg)	72.83 ± 10.76	80.55 ± 11.64	−100.953	<0.001	75.22 ± 10.63	81.11 ± 11.55	−65.368	<0.001	70.00 ± 10.21	77.94 ± 11.68	−50.914	<0.001
FBG (mmol/L)	5.25 ± 0.89	5.90 ± 1.66	−83.434	<0.001	5.37 ± 1.04	5.91 ± 1.68	−50.394	<0.001	5.11 ± 0.65	5.88 ± 1.57	−64.943	<0.001
TC (mmol/L)	4.81 ± 0.91	5.17 ± 0.97	−58.22	<0.001	4.83 ± 0.90	5.15 ± 0.97	−43.13	<0.001	4.78 ± 0.91	5.24 ± 0.99	−35.439	<0.001
TG (mmol/L)	1.30 ± 0.94	2.50 ± 2.13	−128.719	<0.001	1.49 ± 1.10	2.60 ± 2.24	−85.555	<0.001	1.09 ± 0.66	2.04 ± 1.48	−81.758	<0.001
HDL-C (mmol/L)	1.47 ± 0.34	1.21 ± 0.27	123.651	<0.001	1.35 ± 0.30	1.17 ± 0.26	80.032	<0.001	1.61 ± 0.33	1.36 ± 0.29	54.811	<0.001
LDL-C (mmol/L)	2.84 ± 0.79	3.24 ± 0.81	−73.521	<0.001	2.95 ± 0.77	3.23 ± 0.81	−44.67	<0.001	2.73 ± 0.78	3.27 ± 0.83	−48.451	<0.001
UA (mmol/L)	329.41 ± 87.10	407.26 ± 93.55	−130.728	<0.001	379.22 ± 78.21	425.62 ± 87.94	−71.013	<0.001	273.39 ± 57.54	326.43 ± 72.24	−62.804	<0.001
ALT (μ/L)	22.29 ± 22.80	40.06 ± 28.96	−107.389	<0.001	26.66 ± 26.71	42.58 ± 29.96	−71.376	<0.001	17.39 ± 16.04	29.00 ± 20.73	−49.029	<0.001
AST (μ/L)	21.78 ± 15.52	27.32 ± 16.70	−52.224	<0.001	23.33 ± 17.21	28.00 ± 17.65	−33.723	<0.001	20.03 ± 13.15	24.34 ± 11.19	−23.663	<0.001
TBIL (μmol/L)	13.78 ± 5.46	13.78 ± 5.31	−0.018	0.985	14.83 ± 5.81	14.18 ± 5.43	14.333	<0.001	12.59 ± 4.76	12.03 ± 4.35	8.263	<0.001
DBIL (μmol/L)	4.18 ± 1.88	4.03 ± 1.68	12.412	<0.001	4.57 ± 1.89	4.17 ± 1.70	26.45	<0.001	3.74 ± 1.76	3.38 ± 1.43	14.254	<0.001

BMI, body mass index; WC, circumference; SBP, systolic blood pressure; DBP, diastolic blood pressure; FBG, fasting blood glucose; TC, total cholesterol; TG, triglyceride; HDL-C, high-density lipoprotein cholesterol; LDL-C, low-density lipoprotein cholesterol; UA, uric acid; ALT, alanine transaminase; AST, aspartate aminotransferase; TBIL, total bilirubin; DBIL, direct bilirubin.

**Figure 1:**
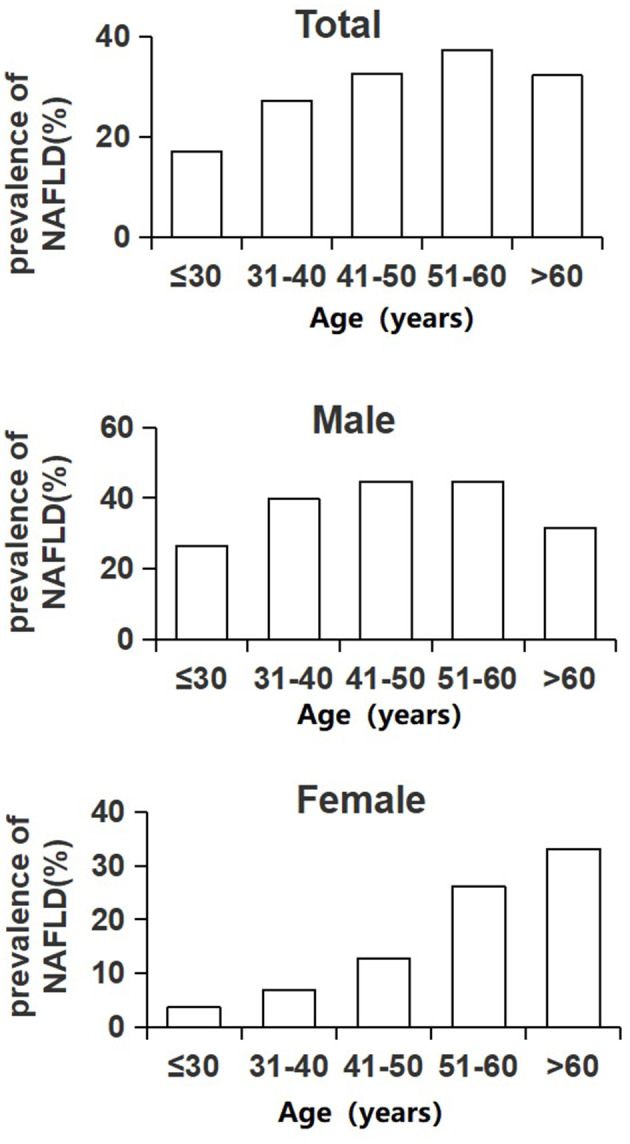
Prevalence of NAFLD in different age groups.

The prevalence rate of NAFLD in individuals in the total population increased with age from 17.0% in younger than 30 years to the peak of 37.3% in people aged between 51 and 60 years and then went down to 32.2% in people older than 60 years. The male population took the same changing trend as the total population, but for the female population, the trend was quite different. As depicted in Figure 1, the prevalence rate of NAFLD for the male population still reached the highest value in the 51–60 years old age group (44.7%), while the prevalence of NAFLD for the female population kept on increasing with age (trend *P* < 0.001). The lowest was 3.7% for women under 30 years, and the highest value was 33.1% for women over 60 years. In each age group below 60 years, the prevalence of NAFLD for men was higher than that of women (*P* < 0.001). However, there was no difference between men and women in the age group of over 60 years (χ^2^ = 3.095, *P* = 0.079) (Figure 1).

### Prevalence of NAFLD in different BMI groups

**Figure 2:**
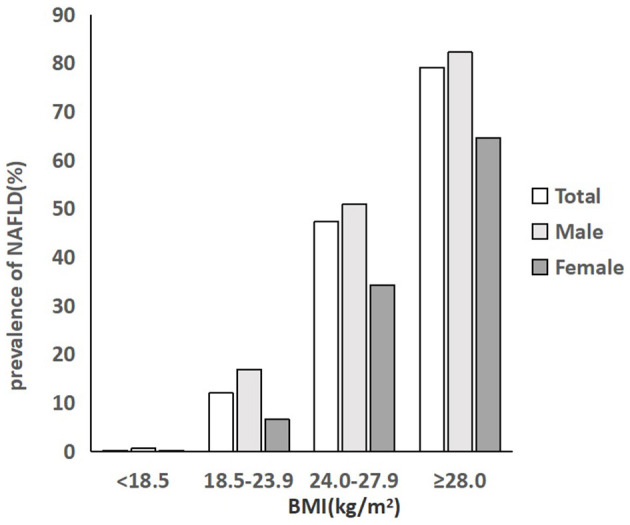
Prevalence of NAFLD in different BMI groups.

As described in Figure 2, the prevalence rate of NAFLD in individuals in the total population, male population and female population, all increased with BMI level. In the total population, 0.2% of people with a BMI below 18.5 kg/m^2^ were diagnosed with NAFLD, and 12% of people with a normal BMI had NAFLD. The rate increased to 47.4% in people with a BMI of 24.0–27.9 kg/m^2^ and then rocketed to the highest level of 79.1% in people with a BMI of over 28.0 kg/m^2^, which was nearly 6.6 times higher than that of normal people. The prevalence of NAFLD in men was significantly higher than that of women in each BMI group (*P* < 0.001). Approximately 50.9% of the men being overweight had NAFLD, which was nearly 1.5 times higher than that of overweight women (34.3%). Among men with obesity, the prevalence rate was 82.2%, much higher than that of women with obesity (64.6%) (Figure 2; Table 2).

**Table 2:** Association between risk factors and NAFLD analyzed by univariable binary logistic regression (*N* = 110,626).

	**Total**				**Male**				**Female**			
	**Non-NAFLD**	**NAFLD**	**OR (95% CI)**	**P-value**	**Non-NAFLD**	**NAFLD**	**OR (95% CI)**	**P-value**	**Non-NAFLD**	**NAFLD**	**OR (95% CI)**	**P-value**
**BMI (Kg/m** ^2^ **)**												
< 18.5	4,649 (99.8)	11 (0.2)	0.02 (0.01–0.03)	<0.001	1,379 (99.4)	8 (0.6)	0.03 (0.01–0.06)	<0.001	3,270 (99.9)	3 (0.1)	0.01 (0.00–0.04)	<0.001
18.5–23.9	47,490 (88.0)	6,451 (12.0)	1.0 (ref)		23,278 (83.1)	4,744 (16.9)	1.0 (ref)		24,212 (93.4)	1,707 (6.6)	1.0 (ref)	
24.0–27.9	17,675 (52.6)	15,921 (47.4)	6.63 (6.41–6.86)	<0.001	12,962 (49.1)	13,461 (50.9)	5.10 (4.90–5.30)	<0.001	4,713 (65.7)	2,460 (34.3)	7.40 (6.91–7.93)	<0.001
≥28.0	1,777 (20.9)	6,728 (79.1)	27.87 (26.29–29.55)	<0.001	1,245 (17.8)	5,758 (82.2)	22.69 (21.19–24.31)	<0.001	532 (35.4)	970 (64.6)	25.86 (23.02–29.06)	<0.001
**Central obesity**												
No	49,724 (90.3)	5,371 (9.7)	1.0 (ref)		23,227 (85.5)	3,935 (14.5)	1.0 (ref)		26,497 (94.9)	1,436 (5.1)	1.0 (ref)	
Yes	21,794 (47.9)	23,727 (52.1)	10.08 (9.75–10.42)	<0.001	15,602 (43.8)	20,031 (56.2)	7.58 (7.28–7.89)	<0.001	6,192 (62.6)	3,696 (37.4)	11.01 (10.30–11.78)	<0.001
**Hypertension**												
No	60,727 (76.6)	18,581 (23.4)	1.0 (ref)		31,499 (67.0)	15,493 (33.0)	1.0 (ref)		29,228 (90.4)	3,088 (9.6)	1.0 (ref)	
Yes	11,115 (51.1)	10,637 (48.9)	3.13 (3.03–3.23)	<0.001	7,457 (46.6)	8,552 (53.4)	2.33 (2.25–2.42)	<0.001	3,658 (63.7)	2,085 (36.3)	5.39 (5.05–5.76)	<0.001
**Glucose status**												
Normal	73,123 (75.0)	24,353 (25.0)	1.0 (ref)		37,702 (65.5)	19,871 (34.5)	1.0 (ref)		35,421 (88.8)	4,482 (11.2)	1.0 (ref)	
IFG	2,179 (43.1)	2,874 (56.9)	3.96 (3.74–4.19)		1,575 (40.4)	2,324 (59.6)	2.80 (2.62–2.99)	<0.001	604 (52.3)	550 (47.7)	7.20 (6.39–8.11)	<0.001
DM	2,426 (38.1)	3,943 (61.9)	4.88 (4.63–5.14)	<0.001	1,906 (37.4)	3,189 (62.6)	3.17 (2.99–3.37)	<0.001	520 (40.8)	754 (59.2)	11.46 (10.20–12.87)	<0.001
**TC (mmol/L)**												
< 5.2	54,582 (76.2)	17,085 (23.8)	1.0 (ref)		28,372 (66.8)	14,093 (33.2)	1.0 (ref)	<0.001	26,210 (89.8)	2,992 (10.2)	1.0 (ref)	
≥5.2	23,466 (62.3)	14,214 (37.7)	1.94 (1.88–1.99)	<0.001	12,939 (53.2)	11,399 (46.8)	1.77 (1.72–1.83)	<0.001	10,527 (78.9)	2,815 (21.1)	2.34 (2.21–2.48)	<0.001
**TG (mmol/L)**												
< 1.70	63,517 (84.3)	11,858 (15.7)	1.0 (ref)		30,552 (77.4)	8,928 (22.6)	1.0 (ref)		32,965 (91.8)	2,930 (8.2)	1.0 (ref)	
≥1.70	14,531 (42.8)	19,441 (57.2)	7.17 (6.96–7.38)	<0.001	10,759 (39.4)	16,564 (60.6)	5.27 (5.09–5.45)	<0.001	3,772 (56.7)	2,877 (43.3)	8.58 (8.07–9.13)	<0.001
**HDL-C (mmol/L)**												
< 1.0	1,582 (34.5)	3,010 (65.5)	5.12 (4.81–5.45)	<0.001	1,337 (32.3)	2,798 (67.7)	3.69 (3.45–3.95)	<0.001	245 (53.6)	212 (46.4)	5.59 (4.64–6.74)	<0.001
≥1.0	75,785 (72.9)	28,137 (27.1)	1.0 (ref)		39,786 (63.8)	22,569 (36.2)	1.0 (ref)		35,999 (86.6)	5,568 (13.4)	1.0 (ref)	
**LDL-C (mmol/L)**												
< 3.40	51,138 (78.8)	13,772 (21.2)	1.0 (ref)		24,971 (69.0)	11,231 (31.0)	1.0 (ref)		26,167 (91.1)	2,541 (8.9)	1.0 (ref)	
≥3.40	26,229 (60.2)	17,375 (39.8)	2.46 (2.39–2.53)	<0.001	16,152 (53.3)	14,136 (46.7)	1.95 (1.89–2.01)	<0.001	10,077 (75.7)	3,239 (24.3)	3.31 (3.13–3.50)	<0.001
**HUA**												
No	65,561 (78.5)	17,974 (21.5)	1.0 (ref)		31,613 (69.5)	13,893 (30.5)	1.0 (ref)		33,948 (89.3)	4,081 (10.7)	1.0 (ref)	
Yes	12,541 (48.5)	13,309 (51.5)	3.87 (3.76–3.99)	<0.001	9,733 (45.6)	11,597 (54.4)	2.71 (2.62–2.80)	<0.001	2,808 (62.1)	1,712 (37.9)	5.07 (4.74–5.43)	<0.001
**ALT (**μ**/L)**												
≤ 40	71,742 (77.8)	20,416 (22.2)	1.0 (ref)		36,115 (69.9)	15,529 (30.1)	1.0 (ref)		35,627 (87.9)	4,887 (12.1)	1.0 (ref)	
>40	6,254 (36.6)	10,839 (63.4)	6.09 (5.88–6.31)	<0.001	5,137 (34.1)	9,928 (65.9)	4.49 (4.32–4.67)	<0.001	1,117 (55.1)	911 (44.9)	5.95 (5.42–6.52)	<0.001
**AST (**μ**/L)**												
≤ 40	74,759 (73.7)	26,658 (26.3)	1.0 (ref)		38,952 (64.6)	21,375 (35.4)	1.0 (ref)		35,807 (87.1)	5,283 (12.9)	1.0 (ref)	
>40	3,237 (41.3)	4,597 (58.7)	3.98 (3.80–4.17)	<0.001	2,300 (36.0)	4,082 (64.0)	3.23 (3.06–3.41)	<0.001	937 (64.5)	515 (35.5)	3.73 (3.33–4.16)	<0.001
**TBIL (**μ**mol/L)**												
≤ 17.1	68,970 (71.2)	27,868 (28.8)	1.0 (ref)		35,201 (61.2)	22,356 (38.8)	1.0 (ref)		33,769 (86.0)	5,512 (14.0)	1.0 (ref)	
>17.1	7,260 (71.2)	2,933 (28.8)	1.00 (0.96–1.05)	0.994	5,284 (66.0)	2,717 (34.0)	0.81 (0.77–0.85)	<0.001	1,976 (90.1)	216 (9.9)	0.67 (0.58–0.77)	<0.001
**DBIL (**μ**mol/L)**												
≤ 6.8	66,057 (70.5)	27,581 (29.5)	1.0 (ref)		34,236 (60.6)	22,228 (39.4)	1.0 (ref)		31,821 (85.6)	5,353 (14.4)	1.0 (ref)	
>6.8	5,019 (76.3)	1,561 (23.7)	0.74 (0.70–0.79)	<0.001	3,736 (71.7)	1,476 (28.3)	0.61 (0.57–0.65)	<0.001	1,283 (93.8)	85 (6.2)	0.39 (0.32–0.49)	<0.001
**Cholelithiasis**												
No	76,067 (72.0)	29,653 (28.0)	1.0 (ref)		40,157 (62.3)	24,266 (37.7)	1.0 (ref)		35,910 (87.0)	5,387 (13.0)	1.0 (ref)	
Yes	3,024 (61.6)	1,882 (38.4)	1.60 (1.50–1.69)	<0.001	1,475 (52.1)	1,358 (47.9)	1.52 (1.41–1.64)	<0.001	1,549 (74.7)	524 (25.3)	2.26 (2.03–2.50)	<0.001

BMI, body mass index; IFG, impaired fasting glucose; DM, diabetes mellitus; TC, total cholesterol; TG, triglyceride; HDL-C, high-density lipoprotein cholesterol; LDL-C, low-density lipoprotein cholesterol; HUA, hyperuricemia; ALT, alanine transaminase; AST, aspartate aminotransferase; TBIL, total bilirubin; DBIL, direct bilirubin.

### Risk factors for NAFLD

As we can draw from the results of univariate binary logistic regression that BMI, central obesity, hypertension, impaired fasting glucose (IFG)/diabetes mellitus (DM), TC, TG, LDL-C, HDL-C, HUA, ALT, AST, DBIL, and cholelithiasis were positively associated with NAFLD risk. From Table 2, we know that people with central obesity, hypertension, and cholelithiasis had 52.1, 48.9, and 38.4% presence of NAFLD compared to 9.7, 23.4, and 28.0% in normal people. When stratified by gender of the participants, all of the aforementioned factors, including TBIL, were risk factors for NAFLD both in the male and female populations (Table 2).

Results of the multivariable logistic analysis, which incorporated age, gender, and all the metabolic factors from Table 2 into the study, showed that gender, age, BMI, central obesity, hypertension, impaired fasting glucose (IFG)/diabetes mellitus (DM), TG, LDL-C, HDL-C, HUA, ALT, and cholelithiasis were all independently correlated with the risk of NAFLD in the whole population (Table 3). Men (OR 1.39) and subjects between the ages of 51–60 years (OR 1.77) were more likely to be diagnosed with NAFLD. Furthermore, NAFLD was more likely to occur in subjects with overweight (OR 2.42), obesity (OR 7.05), central obesity (OR 2.45), as well as those with hypertension (OR 1.37), IFG (OR 1.81), DM (OR 2.42), hyperuricemia (OR 1.75), and cholelithiasis (OR 1.14). People with a high level of TG (OR 2.77), LDL-C (OR 1.35), and ALT (OR 2.79) and a low level of HDL-C (OR 1.64) had a higher possibility of NAFLD. For the male population, age, BMI, central obesity, hypertension, IFG/DM, TG, LDL-C, HDL-C, hyperuricemia, ALT, and cholelithiasis were significantly associated with NAFLD risk. While for the female population, in addition to the aforementioned factors, TC and AST were also independently associated with NAFLD. Obesity was the strongest correlated factor (OR 7.05), both for men (OR 7.08) and women (OR 6.81) (Table 3).

**Table 3:** Association between risk factors and NAFLD analyzed by univariable binary logistic regression (*N* = 110,626).

	**Total**					**Male**					**Female**				
	**B**	**S.E**.	**Wald**	***P***	**OR (95% CI)**	**B**	**S.E**.	**Wald**	***P***	**OR (95% CI)**	**B**	**S.E**.	**Wald**	***P***	**OR (95% CI)**
**Gender**															
Male	0.328	0.023	201.044	<0.001	1.39 (1.33–1.45)										
**Age (years)**			303.644	<0.001				258.211	<0.001				215.247	<0.001	
31–40	0.34	0.032	110.862	<0.001	1.41 (1.32–1.50)	0.335	0.035	92.339	<0.001	1.40 (1.31–1.50)	0.48	0.09	28.23	<0.001	1.62 (1.35–1.93)
41–50	0.424	0.034	160.164	<0.001	1.53 (1.43–1.63)	0.38	0.037	107.322	<0.001	1.46 (1.36–1.57)	0.799	0.088	82.008	<0.001	2.22 (1.87–2.64)
51–60	0.572	0.036	257.607	<0.001	1.77 (1.65–1.90)	0.43	0.04	115.345	<0.001	1.54 (1.42–1.66)	1.143	0.088	168.217	<0.001	3.14 (2.64–3.73)
≥61	0.25	0.04	38.967	<0.001	1.28 (1.19–1.39)	−0.033	0.046	0.519	0.471224	0.97 (0.88–1.06)	1.01	0.094	115.408	<0.001	2.75 (2.28–3.30)
**BMI (Kg/m** ^2^ **)**			3,081.562	<0.001				2,279.725	<0.001				759.009	<0.001	
< 18.5	−2.881	0.303	90.349	<0.001	0.06 (0.03–0.10)	−2.485	0.357	48.569	<0.001	0.08 (0.04-0.17)	−3.224	0.579	30.971	<0.001	0.04 (0.01–0.12)
24.0–27.9	0.885	0.023	1,471.528	<0.001	2.42 (2.32–2.54)	0.846	0.026	1,040.555	<0.001	2.33 (2.21–2.45)	0.978	0.048	411.668	<0.001	2.66 (2.42–2.92)
≥28.0	1.953	0.037	2,726.386	<0.001	7.05 (6.55–7.59)	1.957	0.043	2,077.47	<0.001	7.08 (6.51–7.70)	1.918	0.078	607.888	<0.001	6.81 (5.84–7.93)
**Central obesity**															
Yes	0.897	0.024	1,421.738	<0.001	2.45 (2.34–2.57)	0.87	0.027	1,018.658	<0.001	2.39 (2.26–2.52)	0.893	0.049	335.776	<0.001	2.44 (2.22–2.69)
**Hypertension**															
Yes	0.314	0.023	189.219	<0.001	1.37 (1.31–1.43)	0.302	0.026	137.911	<0.001	1.35 (1.29–1.42)	0.29	0.048	35.969	<0.001	1.34 (1.22–1.47)
**Glucose status**			738.848	<0.001				505.067	<0.001				290.717	<0.001	
IFG	0.596	0.04	227.414	<0.001	1.81 (1.68–1.96)	0.52	0.044	137.319	<0.001	1.68 (1.54–1.83)	0.913	0.084	117.861	<0.001	2.49 (2.11–2.94)
DM	0.885	0.036	591.334	<0.001	2.42 (2.26–2.60)	0.836	0.041	423.299	<0.001	2.31 (2.13–2.50)	1.153	0.081	200.483	<0.001	3.17 (2.70–3.72)
**TC (mmol/L)**															
≥5.2	−0.042	0.027	2.382	0.123	0.96 (0.91–1.01)	0.014	0.031	0.213	0.644284	1.01 (0.96–1.08)	−0.309	0.062	25.23	<0.001	0.73 (0.65–0.83)
**TG (mmol/L)**															
≥1.70	1.02	0.02	2,511.613	<0.001	2.77 (2.67–2.89)	0.946	0.023	1,693.507	<0.001	2.57 (2.46–2.69)	1.177	0.044	717.547	<0.001	3.25 (2.98–3.54)
**HDL-C (mmol/L)**															
< 1.0	0.495	0.043	130.632	<0.001	1.64 (1.51–1.79)	0.485	0.045	114.025	<0.001	1.62 (1.49–1.77)	0.647	0.134	23.19	<0.001	1.91 (1.47–2.48)
**LDL-C (mmol/L)**															
≥3.40	0.304	0.027	126.719	<0.001	1.35 (1.28–1.43)	0.215	0.03	51.42	<0.001	1.24 (1.17–1.32)	0.533	0.061	75.673	<0.001	1.70 (1.51–1.92)
**HUA**															
Yes	0.56	0.021	695.882	<0.001	1.75 (1.68–1.83)	0.472	0.023	411.896	<0.001	1.60 (1.53–1.68)	0.868	0.051	290.102	<0.001	2.38 (2.16–2.63)
Yes	1.027	0.028	1,310.238	<0.001	2.79 (2.64–2.95)	0.971	0.03	1,066.288	<0.001	2.64 (2.49–2.80)	1.217	0.09	183.476	<0.001	3.38 (2.83–4.03)
**Elevated AST**															
Yes	−0.004	0.04	0.009	0.923	1.00 (0.92–1.08)	0.04	0.042	0.882	0.34756	1.04 (0.96–1.13)	−0.281	0.111	6.405	0.011	0.75 (0.61–0.94)
**Elevated TBIL**															
Yes						0.015	0.046	0.108	0.742761	1.02 (0.93–1.11)	0.005	0.128	0.002	0.968	1.01 (0.78–1.29)
**Elevated DBIL**															
Yes	−0.023	0.04	0.318	0.573	0.98 (0.90–1.06)	−0.031	0.057	0.297	0.585508	0.97 (0.87–1.08)	−0.348	0.189	3.407	0.065	0.71 (0.49–1.02)
**Cholelithiasis**															
Yes	0.131	0.042	9.598	0.002	1.14 (1.05–1.24)	0.111	0.051	4.758	0.029157	1.12 (1.01–1.23)	0.183	0.077	5.673	0.017	1.20 (1.03–1.39)

BMI, body mass index; IFG, impaired fasting glucose; DM, diabetes mellitus; TC, total cholesterol; TG, triglyceride; HDL-C, high-density lipoprotein cholesterol; LDL-C, low-density lipoprotein cholesterol; HUA, hyperuricemia; ALT, alanine transaminase; AST, aspartate aminotransferase; TBIL, total bilirubin; DBIL, direct bilirubin.

## Discussion

As the most common cause of liver diseases, NAFLD prevalence, as well as the clinical and economic burden of the disease, have emerged with the increase in individuals with obesity in the population. The prevalence of NAFLD for the overall population varied largely in different countries, and according to some previous research, in America, Netherlands, Italy, South Korea, Japan, and Indonesia, the prevalence rate of NAFLD was 31.3% (19), 22.0% (20), 24.8% (21), 40.2% (22), 18.5% (23), and 51% (24), respectively. In China, the prevalence rate reached 32.9% (95% CI: 28.9–36.8%) in 2018, and over the past two decades, the overall prevalence of NAFLD was estimated to be 29.6% (95% CI: 28.2–31.0%) (25). However, these data were diverse due to the enormous differences in age, regions, customs, lifestyles, and geography in China. An understanding of the epidemiological features of NAFLD in different areas of China is meaningful and necessary. To obtain the epidemiological characteristics of NAFLD in the overall population of Chongqing, a west-central city of China, we conducted a study including ~110 thousand healthy adults.

According to the results of our study, the overall prevalence of NAFLD in Chongqing was 28.5%, which was similar to that of Hong Kong (26) (27%, South China) but lower than Urumqi (27) (54.3%, Northwest China) and higher than Shanghai (28) (15.3%, East China). The difference between our study and others may be related to age range, area variation, dietary habits, and lifestyle differences. The prevalence rate of NAFLD in individuals among the male population (38.1%) was significantly higher than that of the female population (13.6%) (OR = 2.44; 95% CI: 2.31–2.58), indicating that compared to women, men were more likely to be diagnosed with NAFLD. The gender difference in the disease can also be found in a study from Shanghai which revealed that the prevalence of NAFLD was 47.88% for men and 23.28% for women (29). This can be explained by a higher possibility of metabolic syndrome in men and a higher level of estrogen in women. In fact, estrogen plays a protective factor against NAFLD by inhibiting the secretion of proinflammatory cytokines, such as interleukins and tumor necrosis factor-α, which are much related to the accumulation of visceral (30, 31). Deficiency or lower levels of estrogen can cause the increase of these proinflammatory cytokine levels, thus leading to the redistribution of fat tissue and promoting the formation of NAFLD (32).

Our results also revealed that the prevalence of NAFLD changed with age, and it reached the highest level at the age of 51–60 years for the whole population (37.3%) and for the male population (44.7%). For the female population, the prevalence of NAFLD increased with age and reached a peak at over 60 years (33.1%). This was different from that of a Korean study showing that prevalence peaks occurred at 40–49 years in the male population and over 50 years in the female population (33). However, our results were consistent with some other studies (34, 35), which found that the prevalence of NAFLD increased as age increased in women, and the peak prevalence of NAFLD in men was relatively younger than that in women. This gender difference in peak prevalence has not been fully understood. It might also be attributed to estrogen, which drops sharply after menopause [happens around the age of 50 years (36)]. Given that estrogen levels and menopause status were not recorded in our study, this point needs to be confirmed with further evidence and data.

Previous studies have suggested that NAFLD was highly associated with insulin resistance and metabolic syndrome, including obesity, hypertension, type 2 diabetes, and dyslipidemia (7, 37–39). Metabolic syndrome has increasingly become a severe problem in China due to the rapid change in lifestyles. In this study, we found that metabolic factors, such as BMI, central obesity, hypertension, DM, hypertriglyceridemia, and hyperuricemia, were independent risk factors of NAFLD. Insulin resistance is thought to be the key link between metabolic syndrome and nonalcoholic fatty liver disease, and it is also involved in the core pathophysiology of the development of NAFLD (40). Insulin resistance may induce flux of free fatty acids to the liver from the adipose tissues, and it can also cause abnormal lipid storage, lipolysis, and lipid peroxidation, promoting the progression of fatty liver to steatohepatitis and even liver fibrosis (39). Insulin resistance has been acknowledged as the main mechanism of type 2 diabetes, thus, it was not hard for us to find diabetes a risk factor for NAFLD in the current study.

According to the results of our study, the prevalence rate of NAFLD in individuals among the total population, male population and female population, all increased with BMI level. Approximately 47.4% of the people with overweight and 79.1% of the people with obesity had NAFLD, and subjects with obesity had 6.6 times the risk of NAFLD than normal people. These data were much close to an epidemiological study from Japan which revealed the 84% prevalence of NAFLD in those with BMI over 28 kg/m^2^ (41). In addition, we also revealed that people with central obesity were more likely to be diagnosed with NAFLD (52.1 vs. 9.7%). Central obesity, as a measure of visceral adiposity, is more closely associated with insulin resistance and NAFLD. Our findings are consistent with the reported data (29, 35).

The relationship between blood pressure and NAFLD presence was illustrated in previous research (42), and our findings also revealed that hypertension was strongly correlated with NAFLD. Similar to our results, other studies conducted on different kinds of individuals showed that hypertension was significantly associated with the prevalence of NAFLD (29, 43), while the exact mechanism was not fully understood. Accumulating evidence has demonstrated that some common pathophysiological mechanisms exist in both hypertension and NAFLD, including inflammation, renin–angiotensin system–sympathetic nervous system activation, and insulin resistance (44, 45), which may be a reasonable explanation for the close connection between hypertension and NAFLD.

Our study has also shown that in the whole population, TG, LDL-C, and HDL-C were independent risk factors of NAFLD. As has been well known, lipid metabolism plays an important role in the formation of fatty liver. Dyslipidemia will cause the deposition of lipids, especially triglyceride accumulation in the liver. In a sequence, lipid delivery will increase, followed by the exacerbating of hepatic insulin resistance, eventually, the formation of NAFLD (46). This can fully explain that in the current study, total triglyceride was proved to be an independent risk factor of NAFLD. Moreover, total cholesterol (TC) was not correlated with NAFLD for the whole population, and this result was consistent with that of a previous study from Xu et al. (47), but when separated by gender, we found that TC was also an independent risk factor for the female population, implying that male NAFLD incidence may be related to some other confounding factors.

In addition, researchers have revealed that hyperuricemia can produce oxygen-free radical stress and aggravate inflammation, leading to the progression of fatty liver disease (48). Thus, hyperuricemia was much more common in the population with NAFLD, and uric acid has been reported to be of diagnostic value for NAFLD in the previous study conducted in shanghai work units (29). Our results suggest that hyperuricemia is one of the independent risk factors for NAFLD in the individual population of Chongqing, providing another strong evidence for the close association between uric acid and NAFLD.

These findings in our study showed that NAFLD was highly associated with obesity, central obesity, diabetes mellitus, hypertriglyceridemia, and hyperuricemia, which was consistent with the notion that the liver is a target organ of the metabolic syndrome in NAFLD.

Based on some previous reports, elevated serum ALT is associated with oxidative stress in the liver (49) and reflects the severity of liver inflammation (50). Elevated ALT is correlated with the progression of NAFLD, and serum ALT has been used as a marker of liver disease. Our results showed that elevated ALT was closely associated with NAFLD, which was in line with another study conducted on the Chinese population (35).

An early study indicated that cholelithiasis was an independent risk factor of NAFLD in addition to metabolic risk factors and could be regarded as an additional risk factor of liver damage in patients with NAFLD (51). In the present study, a close association between cholelithiasis and NAFLD was also found. We demonstrated that 38.4% of the patients with cholelithiasis had NAFLD compared with 28% of patients without cholelithiasis (*p* < 0.0001). The exact pathophysiology of cholelithiasis presence leading to NAFLD has not been uncovered. Considering the high prevalence of gallstones in patients with central obesity, insulin resistance, and type 2 diabetes (52), we speculated that the association between gallstones and NAFLD might stem from the common pathogenic factors shared by both gallstone and NAFLD. The relationship of cholelithiasis with the risk of NAFLD needs to be proved by further research.

In addition, dietary metabolites or metabolites derived from gut microbiota, such as amino acids, bile acids, and choline, have been revealed in a previous study to have an impact on the progression of NAFLD (53). However, we cannot certify the relationship between them and NAFLD in the present study, and for that, we did not detect or record the content of the above substances. Further data were still needed to make a demonstration of it.

The present study has some limitations. First, our study did not cover enough information, including dietary habits, occupation, exercise, and lifestyle, which may have an important impact on the incidence of NAFLD. Second, an examination of insulin resistance was lacking in the present study. Thus, we cannot fully explain the relationship between metabolic syndrome and NAFLD. Finally, when taking into account the practicability, we employed abdominal US as the diagnosis of NAFLD in our study, which was not accurate in contrast to liver biopsy, the gold-standard test for diagnosis of NAFLD. In general, to make a further and clear understanding of the risk factors of NAFLD, we believe that more large-scale studies are needed.

## Conclusion

The present study, which included 110,626 subjects, explored the prevalence of NAFLD among healthy adults in Chongqing, a west-central city in China. In conclusion, we found that 28.5% of the subjects had NAFLD, and prevalence in men and women was 38.1 and 13.6%, respectively. Men aged 51–60 years and women over 60 years were more likely to be diagnosed with NAFLD. The prevalence of NAFLD in individuals with obesity (BMI ≥ 28.0 kg/m^2^) was 79.1%, compared with 12.0% in subjects with a normal BMI (18.5–23.9 kg/m^2^). The prevalence of NAFLD among people with central obesity was 52.1% compared with 9.7% of normal people. Of the included subjects, 48.9% of people with hypertension had NAFLD, while the prevalence was 38.4% in people with cholelithiasis. Our results from the large sample study in Chongqing once again confirmed that gender, age, BMI, central obesity, hypertension, impaired fasting glucose/diabetes mellitus, TG, LDL-C, HDL-C, HUA, ALT, and cholelithiasis were independent risk factors of NAFLD for the general population. We suggest individuals in this area with higher BMI, WC, higher blood glucose, hypertension, hypertriglyceridemia, hyperuricemia, cholelithiasis, and elevated ALT to pay special attention to NAFLD.

## Data availability statement

The original contributions presented in the study are included in the article/supplementary material, further inquiries can be directed to the corresponding author.

## Ethics statement

The study protocol was approved by Hospital Ethics Committee of the First Affiliated Hospital of Chongqing Medical University. Written informed consent for participation was not required for this study in accordance with the national legislation and the institutional requirements.

## Author contributions

XS designed the study. LK wrote the manuscript. YY was responsible for the collecting and sorting of the data. HL completed the processing of the data. XW and YS were responsible for the modification of the manuscript. All authors listed have made a substantial, direct, and intellectual contribution to the work and approved it for publication.
